# Hypertension care in demographic surveillance sites: a cross-sectional study in Bangladesh, India, Indonesia, Malaysia, Viet Nam

**DOI:** 10.2471/BLT.22.287807

**Published:** 2022-08-22

**Authors:** Pascal Geldsetzer, Min Min Tan, Fatwa ST Dewi, Bui TT Quyen, Sanjay Juvekar, Sayed MA Hanifi, Sudipto Roy, Nima Asgari-Jirhandeh, Daniel Reidpath, Tin Tin Su

**Affiliations:** a Department of Medicine, Stanford University, Stanford, United States of America.; b South East Asia Community Observatory (SEACO), Jeffrey Cheah School of Medicine and Health Sciences, Monash University Malaysia, Jalan Lagoon Selatan, Bandar Sunway, 47500 Selangor, Malaysia.; c Sleman Health and Demographic Surveillance System, Faculty of Medicine, Public Health and Nursing, Universitas Gadjah Mada, Yogyakarta, Indonesia.; d Chililab Health and Demographic Surveillance System, Department of Biostatistics, Hanoi University of Public Health, Hanoi, Viet Nam.; e Vadu Health and Demographic Surveillance System, KEM Hospital Research Centre, Pune, India.; f Institute for Global Health and Development, Queen Margaret University, Edinburgh, Scotland.; g Indian Council of Medical Research, New Delhi, India.; h Asia Pacific Observatory on Health Systems and Policies, WHO Regional Office for South-East Asia, New Delhi, India.

## Abstract

**Objective:**

To determine the proportion of adults with hypertension who reported: (i) having been previously diagnosed with hypertension; (ii) taking blood pressure-lowering medication; and (iii) having achieved hypertension control, in five health and demographic surveillance system sites across five countries in Asia.

**Methods:**

Data were collected during household surveys conducted between 2016 and 2020 in the five surveillance sites in Bangladesh, India, Indonesia, Malaysia and Viet Nam. We defined hypertension as systolic blood pressure ≥ 140 mmHg, diastolic blood pressure ≥ 90 mmHg or taking blood pressure-lowering medication. We defined hypertension control as systolic blood pressure < 140 mmHg and diastolic blood pressure < 90 mmHg. We disaggregated hypertension awareness, treatment and control by surveillance site, and within each site by sex, age group, education, body mass index and smoking status.

**Findings:**

Of 22 142 participants, 11 137 had hypertension (Bangladesh: 211; India: 487; Indonesia: 1641; Malaysia: 8164; and Viet Nam: 634). The mean age of participants with hypertension was 60 years (range: 19–101 years). Only in the Malaysian site were more than half of individuals with hypertension aware of their condition. Hypertension treatment ranged from 20.8% (341/1641; 95% CI: 18.8–22.8%) in the Indonesian site to 44.7% (3649/8164; 95% CI: 43.6–45.8%) in the Malaysian site. Less than one in four participants with hypertension had achieved hypertension control in any site. Hypertension awareness, treatment and control were generally higher among women and older adults.

**Conclusion:**

While hypertension awareness and treatment varied widely across surveillance sites, hypertension control was low in all sites.

## Résumé

**Objectif:**

Déterminer le pourcentage d'adultes souffrant d'hypertension qui ont indiqué: (i) avoir déjà été diagnostiqués hypertendus; (ii) prendre des antihypertenseurs; et enfin, (iii) avoir réussi à garder leur hypertension sous contrôle. Et ce, dans cinq sites de surveillance démographique et sanitaire à travers cinq pays différents en Asie.

**Méthodes:**

Des données ont été récoltées lors d'enquêtes menées auprès des ménages entre 2016 et 2020, sur les cinq sites de surveillance au Bangladesh, en Inde, en Indonésie, en Malaisie et au Vietnam. Nous avons fixé divers critères pour définir l'hypertension: une pression artérielle systolique ≥ 140 mmHg, une pression artérielle diastolique ≥ 90 mmHg ou la prise d'hypertenseurs. L'hypertension était considérée comme sous contrôle lorsque la pression artérielle systolique < 140 mmHg et la pression artérielle diastolique < 90 mmHg. Enfin, nous avons ventilé la sensibilisation à l'hypertension, son traitement et son contrôle selon les sites de surveillance et, pour chaque site, selon le sexe, la tranche d'âge, le niveau d'éducation, l'indice de masse corporelle et un éventuel tabagisme.

**Résultats:**

Sur 22 142 participants, 11 137 souffraient d'hypertension: 211 au Bangladesh; 487 en Inde; 1641 en Indonésie; 8164 en Malaisie; et 634 au Vietnam. L'âge moyen des participants souffrant d'hypertension s'élevait à 60 ans (plage comprise entre 19 et 101 ans). Le site malaisien était le seul site où plus de la moitié des individus hypertendus étaient conscients de leur état. Le traitement de l'hypertension allait de 20,8% (341/1641; IC de 95%: 18,8–22,8%) sur le site indonésien à 44,7% (3649/8164; IC de 95%: 43,6–45,8%) sur le site malaisien. Moins d'un participant sur quatre atteint d'hypertension était parvenu à la maintenir sous contrôle. Les femmes et les adultes plus âgés étaient généralement davantage sensibilisés à l'hypertension, ainsi qu'à son traitement et son contrôle.

**Conclusion:**

D'importantes variations ont été observées en matière de sensibilisation et de traitement de l'hypertension d'un site de surveillance à l'autre. En revanche, quel que soit le site, rares étaient celles et ceux chez qui l'hypertension était sous contrôle.

## Resumen

**Objetivo:**

Determinar el porcentaje de adultos con hipertensión arterial que informaron: (i) haber sido diagnosticados previamente con hipertensión; (ii) tomar hipotensores; y (iii) haber logrado el control de la hipertensión, en cinco sitios del sistema de vigilancia sanitaria sobre la población de cinco países en Asia.

**Métodos:**

Los datos se recopilaron durante las encuestas de hogares que se realizaron entre 2016 y 2020 en los cinco sitios de vigilancia en Bangladesh, India, Indonesia, Malasia y Vietnam. Se definió la hipertensión arterial como una tensión arterial sistólica ≥140 mmHg, una tensión arterial diastólica ≥90 mmHg o la toma de hipotensores. Se definió el control de la hipertensión como una tensión arterial sistólica <140 mmHg y una tensión arterial diastólica <90 mmHg. Se desglosó el conocimiento, el tratamiento y el control de la hipertensión por sitio de vigilancia, y dentro de cada sitio por sexo, grupo de edad, educación, índice de masa corporal y condición de fumador.

**Resultados:**

De los 22 142 participantes, 11 137 tenían hipertensión arterial: Bangladesh: 211; India: 487; Indonesia: 1641; Malasia: 8164; y Vietnam: 634. La edad media de los participantes con hipertensión era de 60 años (rango: 19-101 años). Solo en el sitio de Malasia más de la mitad de las personas con hipertensión eran conscientes de su condición. El tratamiento de la hipertensión osciló entre el 20,8 % (341/1641; IC del 95 %: 18,8-22,8%) en el sitio de Indonesia y el 44,7 % (3649/8164; IC del 95 %: 43,6-45,8%) en el sitio de Malasia. Menos de uno de cada cuatro participantes con hipertensión había logrado el control de ésta en cualquiera de los sitios. El conocimiento, el tratamiento y el control de la hipertensión fueron generalmente mayores entre las mujeres y los adultos mayores.

**Conclusión:**

Mientras que el conocimiento y el tratamiento de la hipertensión arterial variaron en gran medida entre los sitios de vigilancia, el control de la hipertensión fue bajo en todos los sitios.

## ملخص

الغرض تحديد نسبة البالغين المصابين بضغط الدم المرتفع، والذين أبلغوا عن: (1) سبق تشخيص إصابتهم بضغط الدم المرتفع؛ و(2) تعاطي أدوية لخفض ضغط الدم؛ و(3) تحقيق السيطرة على ضغط الدم المرتفع، في خمسة مواقع لنظام المراقبة الصحية والسكانية في خمس دول في آسيا.

الطريقة تم جمع البيانات خلال استطلاعات رأي منزلية أجريت بين عامي 2016 و2020 في مواقع المراقبة الخمسة في بنغلاديش والهند وإندونيسيا وماليزيا وفييت نام. حددنا ضغط الدم المرتفع على أنه ضغط الدم الانقباضي الأكبر من أو يساوي 140 ملم زئبقي، أو ضغط الدم الانبساطي الأكبر من أو يساوي 90 ملم زئبقي، أو تناول أدوية لخفض ضغط الدم. حددنا التحكم في ضغط الدم المرتفع على أنه ضغط الدم الانقباضي الأقل من 140 مم زئبق، وضغط الدم الانبساطي الأقل من 90 مم زئبق. قمنا بتصنيف الوعي بضغط الدم المرتفع، والعلاج والسيطرة حسب موقع المراقبة، وداخل كل موقع حسب الجنس والفئة العمرية والتعليم ومؤشر كتلة الجسم وحالة التدخين.

النتائج من بين 22142 مشاركا، كان 11137 منهم يعانون من ضغط الدم المرتفع: بنغلاديش: 211؛ والهند: 487؛ وإندونيسيا: 1641؛ وماليزيا: 8164؛ وفييت نام: 634. كان متوسط عمر المشاركين المصابين بضغط الدم المرتفع 60 عامًا (المدى: 19 إلى 101 عامًا). فقط في الموقع الماليزي، كان أكثر من نصف الأفراد المصابين بضغط الدم المرتفع على دراية بحالتهم. تراوحت معالجة ضغط الدم المرتفع من %20.8 (341/1641؛ بفاصل ثقة مقداره % 95: 18.8 إلى %22.8)في الموقع الإندونيسي إلى %44.7 (3649/8164؛ بفاصل ثقة مقداره % 95: 43.6-%45.8) في الموقع الماليزي. حقق أقل من واحد من كل أربعة مشاركين مصابين بضغط الدم المرتفع، السيطرة على ضغط الدم المرتفع في أي موقع. كان الوعي بضغط الدم المرتفع، والعلاج، والسيطرة أعلى بشكل عام بين النساء وكبار السن.

الاستنتاج بينما تباين الوعي بضغط الدم المرتفع ومعالجته بشكل كبير عبر مواقع المراقبة، إلا أن التحكم في ضغط الدم المرتفع كان منخفضًا في كل المواقع.

## 摘要

**目的:**

旨在确定在五个亚洲国家所设的五个卫生和人口监测系统站点中，报告存在以下状况的高血压成人患者比例：(i) 以前已被诊断出患有高血压；(ii) 正在服用降血压药物；以及 (iii) 高血压已得到控制。

**方法:**

我们收集了 2016 年至 2020 年期间在马来西亚、孟加拉国、印度、印度尼西亚和越南的五个监测点开展家庭调查所得的数据。我们将收缩压 ≥ 140 mmHg，舒张压 ≥ 90 mmHg，或者正在服用降压药的情况视为高血压。我们将收缩压 < 140 mmHg，舒张压 < 90 mmHg 的情况视为已控制高血压。我们分析了各监测点获知自己患有该疾病、接受治疗、成功控制该疾病的高血压患者分别占多少比例，并在每个监测点内部按性别、年龄、群组、受教育程度、体质指数和吸烟状况统计患者比例。

**结果:**

在 22,142 名参与者中，有 11,137 人患有高血压：孟加拉国：211；印度：487；印度尼西亚：1641；马来西亚：8164；和越南：634。参与者中高血压患者的平均年龄为 60 岁（范围：19–101 岁）。仅在马来西亚监测点即有超过半数的高血压患者知道自己的病情。接受治疗的高血压患者比例，最低为印度尼西亚监测点的 20.8% (341/1641; 95% CI: 18.8–22.8%)，最高为马来西亚监测点的 44.7% (3649/8164; 95% CI: 43.6–45.8%)。无论在哪个监测点，参与者中只有不到四分之一的高血压患者的高血压得到控制。女性和老年高血压患者在了解自己的病情、接受治疗和控制病情方面所占比率普遍较高。

**结论:**

虽然在不同监测点高血压患者在了解自己的病情和接受治疗方面的情况存在很大差异，但所有监测点的高血压控制率均较低。

## Резюме

**Цель:**

Определить долю взрослого населения с гипертензией, которое сообщило: (i) о ранее диагностированной гипертензии; (ii) о приеме препаратов для снижения артериального давления; (iii) о достижении контроля над гипертензией. Сообщения были получены в пяти учреждениях системы здравоохранения и демографического надзора в пяти странах Азии.

**Методы:**

Данные были собраны в ходе обследований домохозяйств, проведенных в период с 2016 по 2020 год в пяти центрах эпиднадзора в Бангладеш, Вьетнаме, Индии, Индонезии и Малайзии. Авторы определили гипертензию как систолическое артериальное давление ≥ 140 мм рт. ст., диастолическое артериальное давление ≥ 90 мм рт. ст. или прием препаратов для снижения артериального давления. Авторы определили контроль гипертензии как систолическое артериальное давление < 140 мм рт. ст. и диастолическое артериальное давление < 90 мм рт. ст. Авторы дезагрегировали осведомленность о гипертензии, ее лечение и контроль по центрам эпиднадзора в рамках каждого центра по полу, возрастной группе, образованию, индексу массы тела и статусу курения.

**Результаты:**

Из 22 142 участников 11 137 страдали гипертензией: Бангладеш: 211; Вьетнам: 634; Индия: 487; Индонезия: 1641; Малайзия: 8164. Средний возраст участников с гипертензией составлял 60 лет (диапазон: 19–101 год). Только в малайзийском центре более половины людей с гипертензией знали о своем состоянии. Показатель лечения гипертензии варьировался от 20,8% (341/1641; 95%-й ДИ: 18,8–22,8%) в индонезийском центре до 44,7% (3649/8164; 95%-й ДИ: 43,6–45,8%) в малайзийском центре. Менее одного из четырех участников с гипертензией добились контроля гипертензии в любом из центров. Осведомленность о гипертензии, ее лечение и контроль в целом были выше среди женщин и пожилых людей.

**Вывод:**

Хотя осведомленность о гипертензии и ее лечение значительно различались в разных центрах эпиднадзора, контроль гипертензии был низким во всех центрах.

## Introduction

We established the Asian Health and Demographic Surveillance Systems Noncommunicable Diseases Network to begin harmonizing data collection efforts and synthesize evidence on noncommunicable diseases across health and demographic surveillance system sites in Asia. Health and demographic surveillance systems are research sites that conduct ongoing detailed demographic and health surveillance of a geographically defined population.^1^ These sites could play an important role in guiding the response of Asian countries to the growing burden of noncommunicable diseases.^2^ Their regular surveillance of an entire geographically defined population could allow for unique and detailed insight into epidemiological and health services changes, including the effects on individuals, households and entire communities. In addition, their routine long-term follow-up and often rich data linkage to health system and administrative data could provide the ideal infrastructure for trialling interventions to prevent or treat noncommunicable diseases.

This joint analysis of the Asian Health and Demographic Surveillance Systems Noncommunicable Diseases Network focuses on the management of hypertension. Hypertension is a major cause of morbidity and mortality globally and increasingly in low- and middle-income countries.^3^ Diagnosing hypertension does not require expensive equipment or a laboratory,^4^ and the condition can be effectively treated using inexpensive medications with few side-effects.^5^^,^^6^ Monitoring and improving the diagnosis and management of hypertension should, thus, be a priority for health systems in low- and middle-income countries.

Understanding where individuals are lost in the care process from diagnosis to successful hypertension control can help inform which step in the care process should be prioritized for improvement. In addition, evidence on which population groups are more likely to achieve each of these care steps could inform the targeting of appropriate interventions. Therefore, this study aimed to determine: (i) the proportion of adults with hypertension living in each of the five health and demographic surveillance systems in our network (located in Bangladesh, India, Indonesia, Malaysia and Viet Nam) who were aware of their hypertension (i.e. had been previously diagnosed), reported taking medication to lower blood pressure, and had achieved control of their hypertension; and (ii) how these proportions varied within the population of each surveillance site by age, sex, body mass index (BMI) and smoking status.

## Methods

### Data sources

The Asian Health and Demographic Surveillance Systems Noncommunicable Diseases Network includes the Chakaria surveillance system (Bangladesh), the Vadu surveillance systems (India), the Sleman surveillance system (Indonesia), the South East Asia Community Observatory (SEACO) surveillance system (Malaysia) and the Chililab surveillance system (Viet Nam). Table 1 gives an overview of the characteristics of each site. A detailed description of each surveillance system has been published separately.^7^^–^^11^ These sites were selected for the Asian Health and Demographic Surveillance Systems Noncommunicable Diseases Network, and hence this analysis because of their interest in engaging in data collection and harmonization efforts on noncommunicable diseases and to ensure coverage of a range of different settings in Asia (e.g. rural and urban areas).

**Table 1:** Overview of the health and demographic surveillance systems and survey sample

Name of system and country	Location	Rural or urban	Surveillance system population^a^	Year(s) of data collection	No. of clusters sampled^b^	Response rate^c^, %	Missing blood pressure^d^, no. (%)	Sample size^e^, no.	Mean age, years	Age range, years	Females, no. (%)	Hypertension, all ages, no. (%)
Chakaria, Bangladesh	Chakaria Upazila, Cox’s Bazar district	Rural	120 000	2019–2020	3	82.0	9 (0.0)	838	38.5	18–101	561 (66.9)	211 (25.2)
Vadu, India	Pune district, Maharashtra	Rural	160 000	2016	22	98.0	11 (0.7)	1504	56.8	31–95	809/1501 (53.9)	487 (32.4)
Sleman, Indonesia	Sleman Regency, Yogyakarta province	Mostly urban	20 000	2018	17	81.6	0 (0.0)	3650	52.4	25–101	2223 (60.9)	1641 (45.0)
SEACO, Malaysia	Segamat district, Johor state	Mostly rural	45 000	2018–2019	5	94.3	16 (0.1)	13 958	57.8	35–99	7913 (56.7)	8164 (58.5)
Chililab, Viet Nam	Chi Linh district, Hai Duong province	Both	60 000	2017	18	95.6	5 (0.2)	2192	47.6	18–70	1287 (58.7)	634 (28.9)

SEACO: South East Asia Community Observatory.

^a^ Approximate number as of January 2019.

^b^ These were villages in the Chakaria, Chililab and Vadu health and demographic surveillance systems, and subdistricts in the SEACO and Sleman surveillance systems.

^c^ Combined household- and individual-level response rate.

^d^ Participants with missing blood pressure measurements.

^e^ All participants who were interviewed (regardless of whether they had hypertension) and did not have missing blood pressure measurements.

Data for this analysis were collected in 2019–2020 in the Chakaria surveillance system, 2016 in the Vadu surveillance system, 2018 in the Sleman surveillance system, 2018–2019 in the SEACO surveillance system and 2017 in the Chililab surveillance system. Each surveillance system, with the exception of the SEACO system, used a two-stage cluster random sampling strategy to select participants. In the first stage, we sampled villages or neighbourhoods (the number sampled in each surveillance system is shown in Table 1) through either simple random sampling or sampling with probability proportionate to population size. Households were sampled in the second stage using systematic random sampling. The SEACO site sampled all households in five of 11 randomly selected subdistricts of the surveillance system. Each surveillance system sampled all adult household members, except for the Sleman site, which sampled (at random) one adult household member per household. The eligible age range for household members was 18 years and older in all five surveillance systems except for the SEACO site (35 years and older) and the Vadu site (30 years and older). During the interview with the participants, each surveillance system measured blood pressure three times in the left upper arm in a seated position using an automated digital blood pressure metre. The data collection teams left at least 1 minute between blood pressure measurements.

All the surveillance systems obtained approval from the relevant local research ethics committees before data collection. Hanoi University of Public Health gave approval for the Chililab surveillance system (017–352/DD-YTCC); the Ethics Review Committee of the International Centre for Diarrhoeal Disease Research, Bangladesh, gave approval for the Chakaria surveillance system (ACT00230); the Monash University Human Research Ethics Committee gave approval for the SEACO surveillance system (2018–13142–45226); the Medical and Health Research Ethics Committee of the Universitas Gadjah Mada gave approval for the Sleman surveillance system (KE/FK/0434/EC/2018); and the KEM Hospital Research Centre Institutional Ethics Committee gave approval for the Vadu surveillance system (KEMHRC/RVM/EC/733).

### Outcome variables

In line with the World Health Organization’s (WHO) HEARTS guideline,^12^ hypertension was defined as having a systolic blood pressure of at least 140 mmHg or a diastolic blood pressure of at least 90 mmHg, or reporting taking blood pressure-lowering medication. As recommended by the WHO STEPwise approach to noncommunicable disease risk factor surveillance manual,^13^ we discarded the first blood pressure measurement and used the mean of the last two blood pressure measurements for each participant to define hypertension. We defined awareness of hypertension status as the participant reporting that they had been told they had hypertension by a health-care worker before the household survey. We defined hypertension treatment as self-reporting to be currently taking blood pressure-lowering medication. Lastly, we defined hypertension control as having a systolic blood pressure < 140 mmHg and a diastolic blood pressure < 90 mmHg. We again used the mean of the last two blood pressure measurements for each participant to define hypertension control. The Chakaria surveillance system did not ask participants whether they had ever been told they had hypertension by a health-care worker; therefore, we excluded this site from the analyses on hypertension awareness.

### Independent variables

We included four sociodemographic characteristics in our analysis – age, sex, education and marital status – as these were collected in all five surveillance systems. We categorized education into four groups: no formal education; at least some primary schooling; at least some secondary schooling; and at least some tertiary education. We categorized marital status into three groups: unmarried; married; and widowed, divorced or separated. Only the Chililab, SEACO and Vadu surveillance systems measured participants’ height and weight and collected data on smoking. We therefore excluded the Chakaria and Sleman sites from the analyses that included BMI or smoking. We categorized BMI into three groups: normal (< 25.0 kg/m^2^); overweight (25.0–29.9 kg/m^2^); and obese (≥ 30.0 kg/m^2^). We defined smoking as reporting ever having smoked cigarettes on a regular basis. 

### Statistical analysis

We calculated the proportion of participants with hypertension who: were aware of their hypertension; were being treated for their hypertension; and had achieved hypertension control. To reach each care step, a participant must have reached the preceding care step, that is, every participant who was treated was considered to also be aware of their hypertension, and participants could not have achieved hypertension control if they were not aware and treated. We calculated these proportions separately for each surveillance system. Within each surveillance system sample, we further disaggregated the data by age group, sex, education, marital status, BMI and smoking status. We used sampling weights to adjust for the survey sampling strategy in each site in all analyses except when showing the sample characteristics. We adjusted standard errors for clustering at the highest sampling unit level: the village for the Chakaria, Chililab, SEACO and Vadu surveillance systems, and the household for the Sleman surveillance system. We used Stata version 13 (StataCorp. LP, College Station, United States of America) for data cleaning and R statistical software version 3.6.0 (R Foundation for Statistical Computing, Vienna, Austria) for statistical analyses.

## Results

### Sample characteristics

Across the five health and demographic surveillance system sites, 22 142 adults participated in the household surveys, of whom 11 137 had hypertension (Table 1). The response rate across the five sites ranged from 81.6% in the Sleman surveillance system to 98.0% in the Vadu surveillance system. The mean age of survey participants varied from 38.5 years in the Chakaria surveillance system to 57.8 years in the SEACO surveillance system. The sample size for our analyses, i.e. the number of participants with hypertension, was largest for the SEACO surveillance system (8164 participants) and smallest for the Chakaria surveillance system (211 participants).

Of the participants with hypertension, 53.6% (5915/11 045) were 60 years and older and 57.0% (6351/11 136) were women (Table 2). Educational attainment among the participants with hypertension varied widely across the surveillance system sites. In the Chakaria and Vadu sites, 50.2% (106/211) and 53.7% (261/486), respectively, of the participants with hypertension had no formal education, whereas this proportion was only 4.4% (28/634) in the Chililab site, 9.6% (778/8079) in the SEACO site and 10.9% (179/1636) in the Sleman site. Of the three sites that measured height and weight, BMI was highest among participants with hypertension in the SEACO surveillance system. About one in four participants with hypertension in the SEACO and Sleman sites reported ever having smoked, 25.3% (2067/8163) and 28.0% (459/1641), respectively, while only 6.5% (30/463) of participants with hypertension in the Vadu surveillance system reported doing so.

**Table 2:** Characteristics of participants in demographic surveillance sites; Bangladesh, India, Indonesia, Malaysia, Viet Nam, 2016–2020

Variable	No. (%)^a^							
	All participants (*n* = 22 142)	All participants with hypertension (*n* = 11 137)		Participants with hypertension in:				
				Chakaria (*n* = 211)	Chililab (*n* = 634)	SEACO (*n* = 8164)	Sleman (*n* = 1641)	Vadu (*n* = 487)
**Age group, years**								
18–29	650 (3.0)	45 (0.4)		24 (11.4)	10 (1.6)	0 (0.0)	11 (0.7)	0 (0.0)
30–39	2482 (11.4)	550 (5.0)		48 (22.7)	40 (6.3)	328 (4.0)	127 (7.7)	7 (1.8)
40–49	4459 (20.4)	1619 (14.7)		42 (19.9)	116 (18.3)	1042 (12.8)	345 (21.0)	74 (18.7)
50–59	5522 (25.3)	2916 (26.4)		54 (25.6)	233 (36.8)	2048 (25.1)	486 (29.6)	95 (24.1)
60–69	5584 (25.6)	3598 (32.6)		26 (12.3)	228 (36.0)	2817 (34.5)	392 (23.9)	135 (34.2)
≥ 70	3133 (14.4)	2317 (21.0)		17 (8.1)	7 (1.1)	1929 (23.6)	280 (17.1)	84 (21.3)
Missing	312 (NA)	92 (NA)		0 (NA)	0 (NA)	0 (NA)	0 (NA)	92 (NA)
**Sex**								
Male	9346 (42.2)	4785 (43.0)		64 (30.3)	339 (53.5)	3515 (43.1)	652 (39.7)	215 (44.2)
Female	12 793 (57.8)	6351 (57.0)		147 (69.7)	295 (46.5)	4649 (56.9)	989 (60.3)	271 (55.8)
Missing	3 (NA)	1 (NA)		0 (NA)	0 (NA)	0 (NA)	0 (NA)	1 (NA)
**Marital status **								
Single	1174 (5.3)	483 (4.4)		10 (4.7)	19 (3.0)	394 (4.8)	57 (3.5)	3 (0.7)
Married	16 434 (74.8)	8185 (74.0)		168 (79.6)	518 (81.7)	5995 (73.4)	1238 (75.5)	266 (63.6)
Widowed, divorced or separated	4373 (19.9)	2398 (21.7)		33 (15.6)	97 (15.3)	1774 (21.7)	345 (21.0)	149 (35.6)
Missing	161 (NA)	71 (NA)		0 (NA)	0 (NA)	1 (NA)	1 (NA)	69 (NA)
**Education**								
No formal education	2582 (11.8)	1352 (12.2)		106 (50.2)	28 (4.4)	778 (9.6)	179 (10.9)	261 (53.7)
At least some primary schooling	7178 (32.7)	4319 (39.1)		86 (40.8)	96 (15.1)	3662 (45.3)	437 (26.7)	38 (7.8)
At least some secondary schooling	10 584 (48.3)	4791 (43.4)		18 (8.5)	438 (69.1)	3363 (41.6)	810 (49.5)	162 (33.3)
At least some tertiary education	1585 (7.2)	584 (5.3)		1 (0.5)	72 (11.4)	276 (3.4)	210 (12.8)	25 (5.1)
Missing	213 (NA)	91 (NA)		0 (NA)	0 (NA)	85 (NA)	5 (NA)	1 (NA)
**BMI, kg/m^2^**								
< 25 (normal)	7324 (42.8)	2830 (31.7)		NC^b^	470 (74.2)	2104 (26.9)	NC^b^	256 (55.3)
25 to < 30 (overweight)	5849 (34.1)	3431 (38.5)		NC^b^	154 (24.3)	3122 (39.9)	NC^b^	155 (33.5)
≥ 30 (obese)	3959 (23.1)	2657 (29.8)		NC^b^	9 (1.4)	2596 (33.2)	NC^b^	52 (11.2)
Missing	522 (NA)	367 (NA)		NA	1 (NA)	342 (NA)	NA	24 (NA)
**Ever smoked**								
No	14 108 (74.1)	7711 (75.1)		NC^b^	NC^b^	6096 (74.7)	1182 (72.0)	433 (93.5)
Yes	4933 (25.9)	2556 (24.9)		NC^b^	NC^b^	2067 (25.3)	459 (28.0)	30 (6.5)
Missing	71 (NA)	25 (NA)		NA	NA	1 (NA)	0 (NA)	24 (NA)

BMI: body mass index; NA: not applicable; NC: not collected.

^a^ We calculated percentages based on all non-missing observations for each variable.

^b^ Data not collected at site.

### Hypertension care measures

**Fig. 1:**
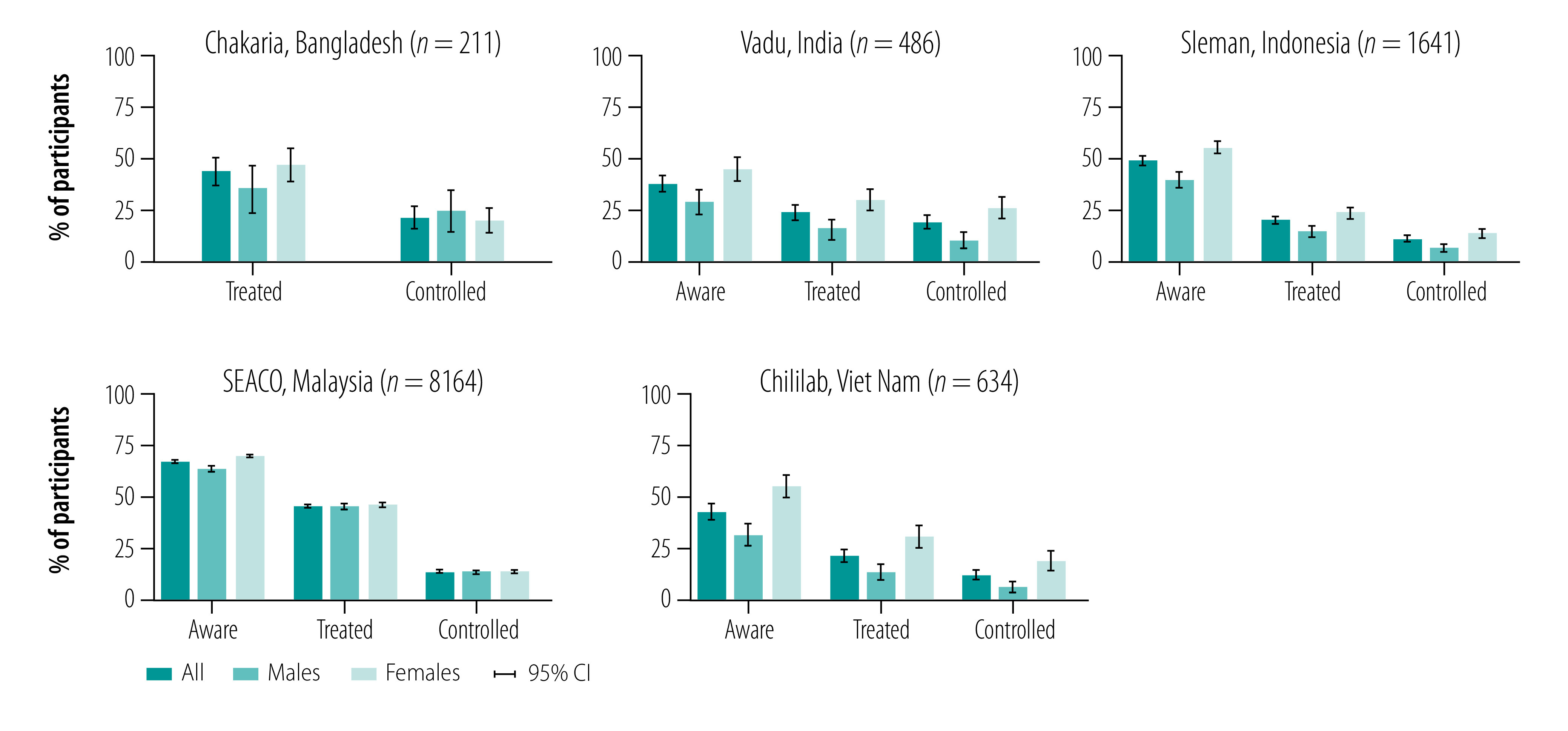
Hypertension awareness, treatment and control among participants with hypertension, by sex in the five health and demographic surveillance systems

Participants’ awareness of their hypertension was highest in the SEACO surveillance system, where 66.0% (5391/8164; 95% confidence interval, CI: 65.0–67.1) of the participants reported having been diagnosed, compared with 49.9% (819/1641; 95% CI: 47.5–52.4) in the Sleman, 44.2% (280/634; 95% CI: 40.3–48.0) in the Chililab and 36.8% (179/486; 95% CI: 32.5–41.1) in the Vadu surveillance systems (Fig. 1). At 44.7% (3649/8164; 95% CI: 43.6–45.8) and 42.7% (90/211; 95% CI: 36.0–49.3), respectively, the proportion of treated participants for hypertension in the SEACO and Chakaria surveillance systems was about twice as high as the proportion in the Sleman (20.8% (341/1641); 95% CI: 18.8–22.8), Vadu (22.8% (111/486); 95% CI: 19.1–26.5) and Chililab (23.0% (146/634); 95% CI: 19.8–26.3) surveillance systems. The proportion of participants achieving hypertension control, however, was low across all five surveillance system sites, ranging from 11.5% (189/1641; 95% CI: 10.0–13.0) in the Sleman site to 20.4% (43/211; 95% CI: 14.9–25.8) in the Chakaria site. Female participants with hypertension were more likely to be aware of and treated for their hypertension and have achieved hypertension control in each surveillance system site. An exception was the SEACO site for treatment and hypertension control and the Chakaria site for hypertension control.

CI: confidence interval.

Note: The Chakaria health and demographic surveillance system did not collect data on hypertension awareness.

### Variation across population groups

**Fig. 2:**
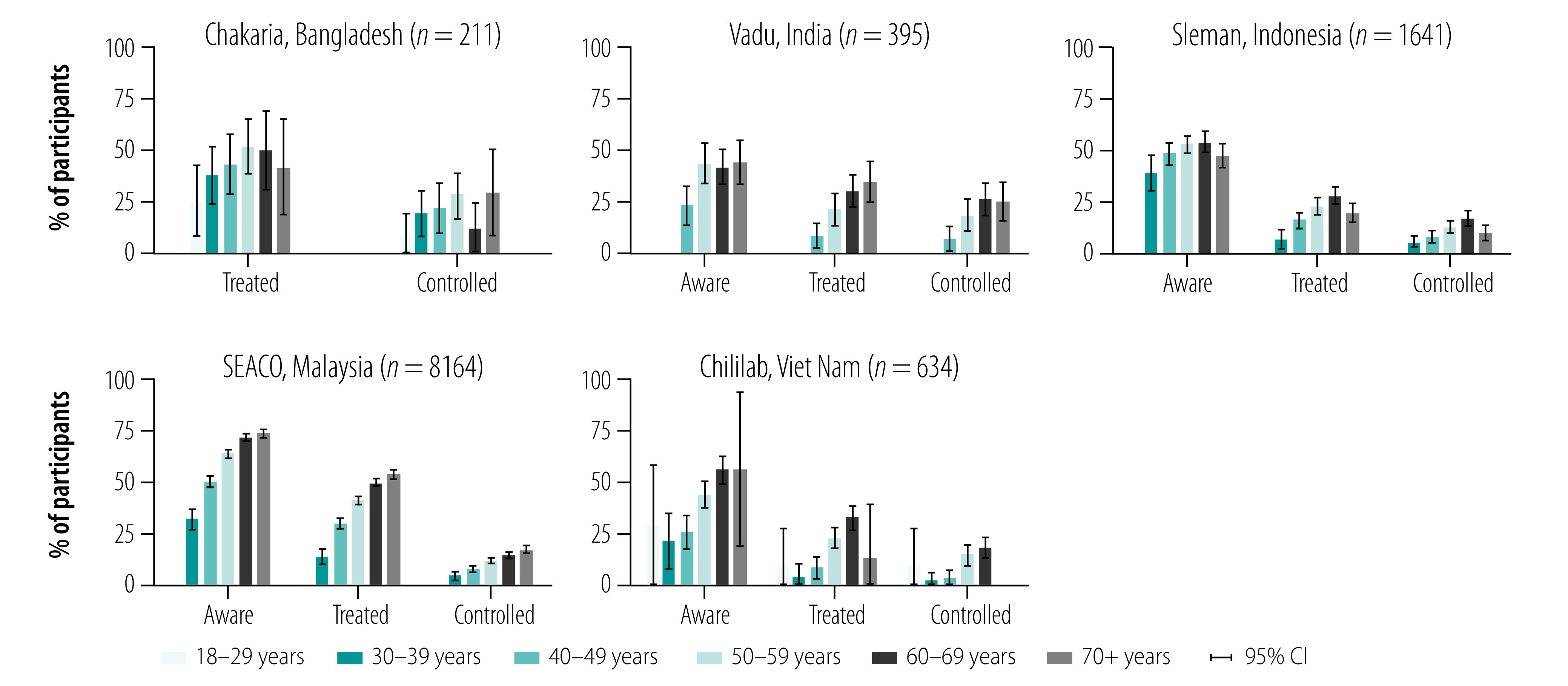
Hypertension awareness, treatment and control among participants with hypertension, by age group in the five health and demographic surveillance systems

Although there was some variation across surveillance systems, older age groups tended to have a higher proportion of awareness, treatment and control of their hypertension (Fig. 2). Of our five surveillance system sites, the absolute differences in the proportion of awareness, treatment and control between different age groups tended to be greatest in the SEACO surveillance system. We saw no clear pattern in hypertension awareness, treatment and control by educational attainment or by BMI (available from the data repository).^14^ Participants with hypertension who reported never having smoked tended to be more aware of their hypertension, being on treatment and achieving control (data repository),^14^ particularly in the Sleman and Vadu sites. Generally, the absolute differences in hypertension awareness, treatment and control by educational attainment, BMI and smoking status were substantially smaller than those by age group.

CI: confidence interval.

Note: The Chakaria health and demographic surveillance system did not collect data on hypertension awareness.

## Discussion

Hypertension control was low in all five sites in the Asian Health and Demographic Surveillance Systems Noncommunicable Diseases Network. However, we saw important variation in the proportion of hypertension awareness and treatment across the sites. Notably, with two thirds of participants with hypertension being aware of their condition, the population in the SEACO surveillance system has a similar proportion of hypertension awareness as has been estimated for some high-income countries.^15^ We also observed large differences in hypertension awareness, treatment and control by age group within each surveillance system site, with older individuals having a substantially higher level of awareness, treatment and control. Overall, the prevalence of hypertension diagnosis, treatment and control in our five sites, and their wide variation across sites, is broadly similar to rates reported from nationally representative surveys in low- and middle-income countries.^16^

Our study also showed a gender difference in hypertension care in our surveillance system sites. Despite having a higher prevalence of hypertension, male participants were less likely than female participants to reach each step of the hypertension care pathway. This discrepancy by sex has been previously reported in low- and middle-income countries.^16^^,^^17^ It is, in fact, an observation that is not unique to hypertension but appears to apply to chronic disease care more broadly, including human immunodeficiency virus infection and diabetes.^18^^–^^23^ The reasons for this discrepancy may include: the fact that blood pressure measurements are a core component of antenatal care visits;^24^ that in general men use health-care services less often than women and thus have fewer chances for opportunistic screening for hypertension by the health system;^25^ and that gendered social norms and cultural beliefs discourage men from seeking preventive services.^26^

Given their population-based longitudinal design, health and demographic surveillance systems are a unique resource for monitoring the rise of noncommunicable diseases in low- and middle-income countries and establishing the population-level effects of epidemiological changes and interventions. Population-based cohort studies are able to determine individual-level risk factors of noncommunicable diseases. However, unless such studies are truly representative of an entire population, they are unable to determine what changes at the population level drive population-level changes in noncommunicable disease epidemiology. An additional unique advantage of health and demographic surveillance systems is that their data can be used to assess so-called spillover effects of epidemiological changes and health service interventions on entire households and communities.^27^^,^^28^ For example, the prescription of antihypertensive medications to individuals may affect the behaviour of their household members or neighbours through various channels. Seeing others obtain free medications that prevent adverse health outcomes may influence household members or neighbours to visit a health care facility for hypertension screening. Conversely, being asked to take medications despite not having any symptoms may dissuade these individuals from seeking hypertension screening. These spillover effects, which can be substantial,^29^ are usually ignored in studies that focus on individual patients or cohort study participants. Furthermore, given that health and demographic surveillance systems conduct routine follow-up of their surveillance population, they may also be an ideal setting for trialling interventions because ascertainment of the long-term outcome is assured.

Our study has some limitations. First, we conducted a cross-sectional rather than a longitudinal assessment of hypertension care. Each step of the care process therefore includes a different set of individuals who are unlikely to be exchangeable with each other. As such, we cannot assume that the proportion of people who achieved hypertension control among those diagnosed would remain the same if all those with hypertension were diagnosed. In addition, the cross-sectional nature of our study prevented us from determining how long individuals take to transition from diagnosis to treatment and control, and to what degree they move backward, i.e. from being controlled to becoming uncontrolled or being treated to being untreated. A recent analysis showed that such backward movement is common for hypertension care in China, Indonesia, Mexico and South Africa.^30^ Second, despite our efforts to harmonize the data, typically by mapping similar questions and grouping similar answers, variation in data collection methods (e.g. how participants were sampled and how questions were asked and responses categorized) across sites could account for some of the variation between the sites that we observed in this analysis. We see the Asian Health and Demographic Surveillance Systems Noncommunicable Diseases Network as an important first step to improved harmonization of data collection efforts and collaboration across health and demographic surveillance systems in Asia. Third, health and demographic surveillance systems only survey a geographically restricted population. As such, they are not designed to be representative of an entire country’s population.^1^ Similarly, while the countries in which our five surveillance systems are located represent 61% of the population in Asia outside of China (and 42% when China is included),^31^ they are not representative of Asia as a whole. Therefore, our results should be interpreted as applying to the specific setting in which each surveillance system is located. Lastly, the surveys were not all conducted in the same year. Time trends in hypertension awareness, treatment and control may therefore explain some of the differences that we observed between surveillance system sites.

In conclusion, this first collaborative analysis of the Asian Health and Demographic Surveillance Systems Noncommunicable Diseases Network found that hypertension control is low across all five health and demographic surveillance systems. However, the proportions of participants being aware of their hypertension and treated vary widely between sites and among population groups within each site. Health and demographic surveillance systems in general, and our collaboration in particular, could serve as a unique resource to monitor the rise of noncommunicable diseases in Asia and to assess the population-level effects of epidemiological changes and health service interventions.
