# Corrigendum

**DOI:** 10.2471/BLT.23.100523

**Published:** 2023-05-01

In: Geldsetzer P, Tan MM, Dewi FST, Quyen BTT, Juvekar S, Hanifi SMA, et al. Hypertension care in demographic surveillance sites: a cross-sectional study in Bangladesh, India, Indonesia, Malaysia, Viet Nam. Bull World Health Organ. 2022 Oct 1;100(10):601–609, 

On page 601, the affiliation of Sayed MA Hanifi should be: Health Systems and Population Studies Division, icddr,b, Dhaka, Bangladesh.

